# The intertidal polychaete (Annelida) fauna of the Sitakunda coast (Chittagong, Bangladesh), with notes on the Capitellidae, Glyceridae, Lumbrineridae, Nephtyidae, Nereididae and Phyllodocidae of the “Northern Bay of Bengal Ecoregion”

**DOI:** 10.3897/zookeys.419.7557

**Published:** 2014-06-23

**Authors:** Alexander I. Muir, Md. M. Maruf Hossain

**Affiliations:** 1 The Natural History Museum, London SW7 5BD, United Kingdom; 2 Institute of Marine Sciences and Fisheries, University of Chittagong, Bangladesh

**Keywords:** Taxonomy, Polychaeta, new records, keys, Odisha, West Bengal, Myanmar

## Abstract

Of seven species of polychaetous annelids collected from the intertidal zone of Sitakunda coast, Chittagong, Bangladesh, five were new records for the country. The seven are listed, with brief notes on these, some previously recorded! species and others housed in the collections of the Natural History Museum, London. Keys are given to the recorded species of Phyllodocidae, Nereididae, Lumbrineridae, Nephtyidae and Capitellidae of the “Northern Bay of Bengal Ecoregion”, and to the recognised species of Glyceridae from the Bay of Bengal. The worms in this Ecoregion are subject to the outflows of the Irrawaddy, Ganges, Hooghly and Mahanadi Rivers, and many of them are known to be freshwater tolerant.

## Introduction

There has long been an emphasis on taxonomy in marine studies, for example Hedgpeth (1957) recommends that the first procedure in any ecological works or applied research with organisms is the exercise of systematics. No ecological investigation can be successfully carried out without a comprehensive knowledge of the taxonomy of faunal resources.

Polychaete annelids are a major group within the soft bottom macro-invertebrates (Gray and Elliott 2009) and comprise a diverse, abundant and ecologically significant functional component of the coastal ecosystem (Misra 1999). These worms are pivotal parts of food webs and form the central link between the sediment systems and higher predators. They are often diverse and highly abundant, especially in areas of anthropogenic stress (Gray and Elliott 2009) and they have diverse feeding strategies (Fauchald and Jumars 1979).

The polychaete fauna of Bangladesh is little studied, despite the importance of marine resources to the country. The largest identification works for the littoral and shallow-water polychaetes of the Indian Ocean area are Fauvel (1953) for the Persian Gulf to Myanmar and Day (1967) for southern Africa. Hartman (1974a, 1974b) is more concerned with deep water polychaetes. There have been many smaller publications on the polychaetes of India, Thailand and Ceylon/Sri Lanka since Fauvel (1953), but only a few for Bangladesh (Mahmood et al. 1993, Belaluzzaman 1995, Alam et al. 1996, Das and Reynolds 2003, Pramanik et al. 2009) and even fewer for Myanmar (one new species each in Kirtley 1994 and Glasby 1999, one re-described species in Böggemann 2002). The most relevant recent publications are probably Misra (1999) and Pramanik et al. (2009).

The present study therefore aims to provide further information on the taxonomy of polychaetes in Bangladesh waters at two sites on the Sitakunda Upazila coast, north of the city of Chittagong (see Table 1), one of which is affected by ship-breaking activity on the shore.

**Table 1.** Details of the sampling sites.

Site	Name & location	Substratum	Remarks
1	Muradpur, 22°35'02"N, 91°34'09"E	Silty-muddy with fine grain of sand (towards sea side)	Relatively undisturbed site along with planted mangroves (relatively high Organic Carbon and Organic Matter compared to other site)
2	Madambabirhat, 22°30'56"N, 91°43'44"E	Sandy cum muddy	Highly polluted & disturbed area due to Ship Breaking Activities in intertidal zone of the coast (low OC & OM)

**Figure 1.**
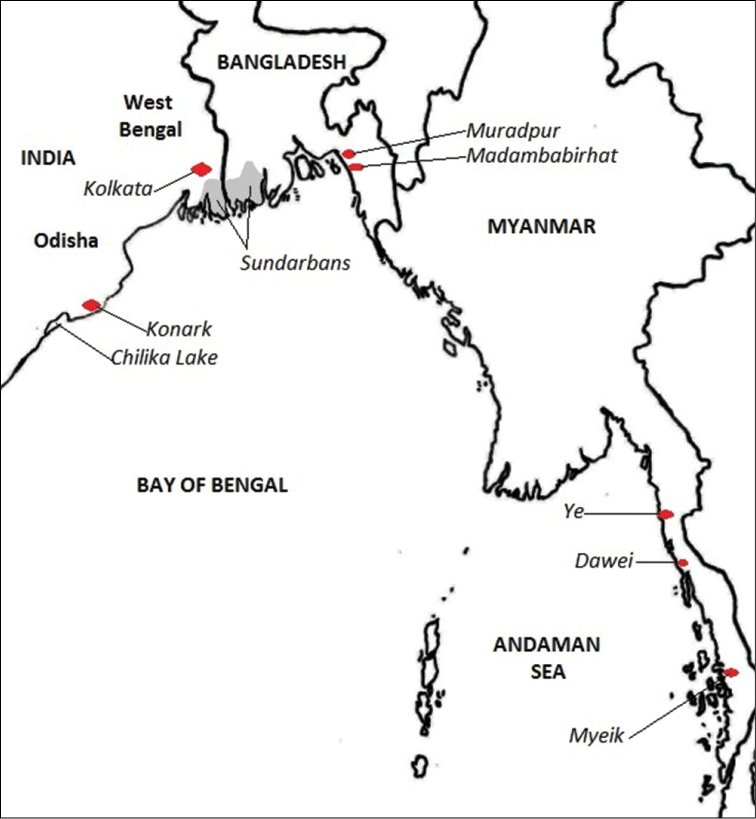
Map of localities mentioned in the text.

The “Northern Bay of Bengal Ecoregion” of the “Bay of Bengal Province” of the “Western Indo-Pacific Realm” was devised by Spalding et al. 2007, and it is shown in map form in Claus et al (2014). The ecoregion extends from between Ye and Dawei (14.61°N, 97.90°E) in Myanmar/Burma to near Konark (19.87°N, 86.11°E) in Odisha/Orissa, India (Fig. 1), and reaches from the coastline to 370km offshore (or the 200m isobath if this is further offshore). It thus includes the Gulf of Martaban, the mouths of the Irrawaddy, Ganges and Hooghly Rivers, and most of the mouths of the Mahanadi River (one distributary leads to the Chilika Lake, usually referred to as Chilka Lake, which has its outlet to the sea in the neighbouring “Eastern India Ecoregion” of the “Bay of Bengal Province”). Southern Myanmar is in the “Andaman Sea Coral Coast Ecoregion” of the “Andaman Province”.

In this paper, the new specimens from Bangladesh are compared with the same families of polychaetes reported from the “Northern Bay of Bengal Ecoregion”, including the entire coast of Myanmar and the entire Odisha coast (to include the freshwater polychaetes of Chilka Lake). Important localities are shown in Figure 1.

## Methods

Quantitative samples were collected between April 2007 and November 2008, but the present paper only deals with the taxonomic details of polychaetes collected at those sites. Samples were collected from the intertidal zone by using a hand-held corer with a depth penetration of 15 cm. The collected samples were washed through a 0.5 mm mesh hand sieve with filtered water at the collection point to separate animals from sediment. The materials retained on the sieve were placed in plastic vials to which 5% formalin was added for fixing the organisms, and labelled. The vital stain Rose Bengal was added to the vials to help in sorting the organisms from debris. In the laboratory the materials were poured into a round transparent Petri dish and separated from debris using needle, brush and magnifying glass. Then the organisms were preserved in 75% ethyl alcohol for identification. An Olympus compound microscope with video facility was used and relevant keys (Fauvel 1953, Day 1967) were followed for preliminary identification. Identification to species necessitated the use of many other papers, which are mentioned later in this publication.

Because there are so few records from Myanmar, some specimens deposited in the Natural History Museum, London, by Professors G.E. Gates (Judson College, Rangoon) and F.J. Meggitt (University College, Rangoon) between 1931 and 1938, and only partially published by C.C.A. Monro (1931, 1937), have been re-studied.

Identification keys are given in this paper, but any identifications made using them should be checked against good descriptions or reliably identified specimens, because not only may new records or even new species be found, but some of the older reports cited here may have been mis-identifications or represent cryptic species (it is interesting that the type locality of *Capitella capitata* is West Greenland (Blake 2009), and for *Glycera alba* is Norway (Böggemann 2002)).

## Taxonomy

### Annelida

The taxonomy and systematics of the Annelida have been rapidly changing in recent years. It must be recognised that the classifications used in publications such as Fauvel (1953) and Day (1967) are now very dated. The fauna given in Fauvel (1953) shares many species with his earlier work on the fauna of France (Fauvel 1923, 1927), but it is not now considered likely that so many species from northern Europe would also be found in the Indian Ocean. A more modern classification (although still on classical lines) can be found in Chambers and Muir (1997). More strictly phylogenetic classifications are also available, such as Rouse (2000) and Appeltans et al. (2010). Keys to identify polychaetes to family level can be found in publications such as Fauchald (1977), Chambers and Muir (1997) and Glasby and Fauchald (2000).

Polychaetous annelids are often regarded as a marine group (albeit with some freshwater tolerant species), but it should be noted that non-marine species also exist (see Glasby et al. 2009), including some from Bangladesh (Das and Reynolds 2003 list two species of *Aeolosoma*).

### Phyllodocidae
*Eteone* cf. *delta* Wu & Chen, 1963

**Figure 2.**
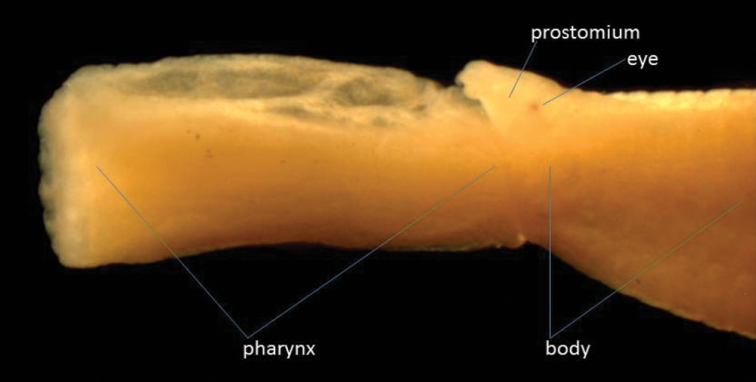
Lateral view of anterior end of *Eteone* cf. *delta* with pharynx extended.

One specimen was found: length 15 mm, width 0.75 mm for 92 segments, but anal cirri missing. Anteriorly the height of the segments is 1mm, but posteriorly the body becomes dorso-ventrally flattened. This specimen has two pairs of tentacular cirri on the first segment, the dorsal being shorter than the ventral ones (they are both, however, small and difficult to see). The first chaetae are on the second segment. The pharynx is everted, showing a smooth surface and a ring of 12 large subglobular papillae around the opening (Fig. 2). The dorsal cirri are small and rounded, compressed against the side of the body. The ventral cirri, distally rounded, are almost as long as the chaetal lobe anteriorly, but slightly longer posteriorly. The tip of the acicula is just emergent from the chaetal lobe in the anterior part of the body, but in the posterior part of the body is much more protuberant. The specimen is colourless in alcohol except for some brown markings dorsally by the pygidium.

This specimen, especially the structure of the pharynx, displays similarities to *Eteone delta* Wu & Chen, 1963, which is known from the Yangtze delta, the Pearl River and Zhangjiang estuary, China (Shen and Qi 1982, Chen et al. 2012). There are, however, differences such as the presence of emergent acicula. It is not considered advisable to describe this specimen as a species new to science, partly because there is only one specimen and partly because that specimen is incomplete (the shape of the anal cirri is important at the generic level for this group). It may be that this is a rare species which shows some morphological variation from one extreme of its geographic range to the other. Glasby et al. (2009) list *Eteone delta* as freshwater tolerant, found in the Palaearctic and Oriental regions inhabiting lake/river freshwater and estuary and coastal lagoons (fresh–brackish) including supra-littoral areas. Shen and Qi (1982) say it is “favored in normal or rich trophic waters”, as opposed to over-trophic or polluted waters.

This is a new record for Bangladesh, no members of the family Phyllodocidae being recorded by Pramanik et al. (2009).

### Discussion of Northern Bay of Bengal Phyllodocidae

Two specimens from Maungmagaun, Myanmar, in the Natural History Museum, London, (NHMUK ANEA 1935.1.31.34 and NHMUK ANEA 1937.1.4.4) have been identified as *Phyllodoce castanea* by C.C.A. Monro. On both of these specimens many of the head appendages are missing or regenerating, but the identifications are probably correct. The species is now known as *Nereiphylla castanea* (see synonymy in Alós et al. 2004). Fauvel (1932) records *Phyllodoce madeirensis* and *Eulalia (Pterocirrus) magalhaensis* from a depth of 2 fathoms (3.658 m) in the Mergui Archipelago.

Two species of *Eteone* are recorded from West Bengal (Misra 1999, Das et al. 2009, Mitra and Misra 2010). *Eteone barantollae* Fauvel, 1932, is now regarded as a member of the genus *Hypereteone* (see Wilson 1988). *Eteone ornata* Grube, 1878, has been referred to the genus *Mysta*, but may be a misidentification (Uschakov, in Wilson 1988).

In Odisha, *Anaitides madeirensis*, *Eteone (Mysta) ornata* and *Eteone barantollae* have been recorded from estuaries by Misra (1999) and Mitra et al. (2010). *Anaitides madeirensis* is now generally referred to as *Phyllodoce madeirensis*, and has a very wide distribution in temperate and tropical waters (Alós 2004).

These species from northern Bay of Bengal waters can be keyed out as follows, but any identifications must be checked against reliable descriptions as many other species are known from the Indo–Pacific area.

**Table:** 

1	Two pairs of tentacular cirri	2
–	Four pairs of tentacular cirri	4
2	Pharyngeal surface smooth, but terminates in a ring of 12 large subglobular papillae	*Eteone* cf. *delta* Wu & Chen, 1963
–	Pharyngeal surface distally with rows of swollen papillae	3
3	Pharynx with five distal rows of swollen papillae	*Hypereteone barantollae* (Fauvel, 1932)
–	Pharynx with three to four rows of swollen papillae. Body with three rows of dark spots	*Mysta ornata* Grube, 1878
4	Median antenna present on head	*Eulalia magalaensis* Kinberg, 1866
–	Median antenna absent	5
5	Segments 1 and 2 fused, but not forming a collar; Pharynx with small, irregularly distributed, papillae	*Nereiphylla castanea* (Marenzeller, 1879)
–	Segment 1 covered dorsally by the posterior part of the prostomium, but not fused to segment 2; Pharynx with 12 longitudinal rows of papillae proximally and 6 rugose bands distally	*Phyllodoce madeirensis* Langerhans, 1880

### Nereididae

The pharynx is often not everted in preserved material, but the jaws and any paragnaths/papillae present may be seen by making a mid-ventral cut backwards from the mouth, cutting through the ventral surface of the pharynx as well as the body wall for several segments, and folding the resulting flaps to the side to reveal the complete jaw apparatus.

### *Neanthes chingrighattensis* (Fauvel, 1932)

**Figure 3.**
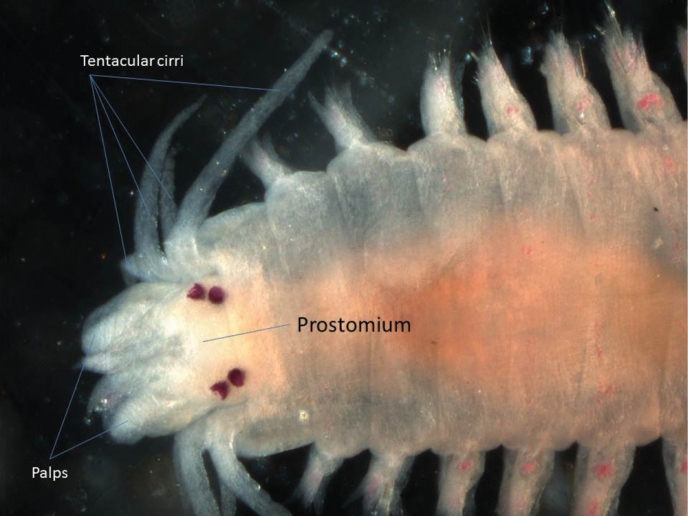
Dorsal view of anterior end of *Neanthes chingrighattensis*.

One specimen was found. This species could be regarded as a typical nereidid, having paragnaths on the pharynx and four pairs of tentacular cirri (Fig. 3). The arrangement of the paragnaths agrees with that depicted by Fauvel (1953). Falcigerous chaetae are entirely absent in this species. It is a new record for Bangladesh according to Pramanik et al. (2009). The type locality is Kolkata, West Bengal, and Misra (1999) states that the species is endemic in Indian waters.

### *Lycastonereis indica* Rao, 1981

**Figure 4.**
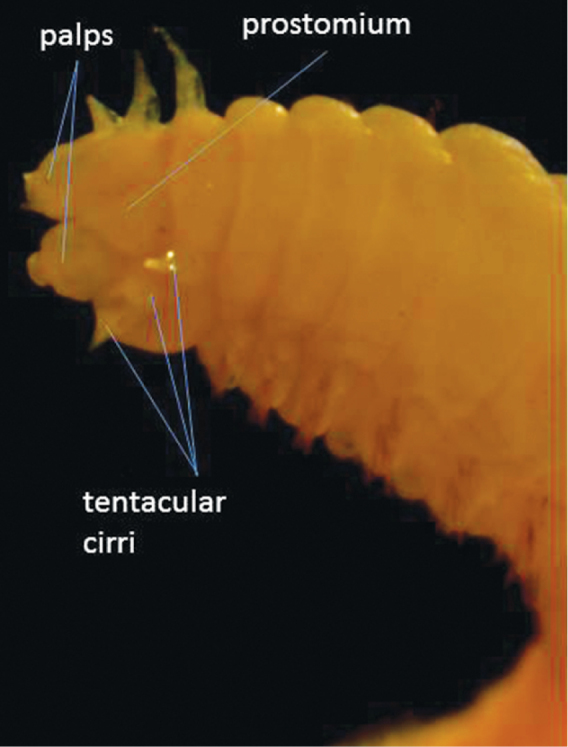
Dorsal view of anterior end of *Lycastonereis indica*.

This species has no paragnaths on the pharynx and only three pairs of tentacular cirri (Fig. 4). It is, however, not a member of the genus *Namanereis* because it has parapodia with two distinct branches (notopodium and neuropodium) each with chaetae. Members of *Namanereis* only have one parapodial lobe with chaetae. This species is not mentioned in Pramanik et al. (2009) but is recorded by Alam et al. (1996) as common on the Halishahar Coast and Misra (1999) states that the species is endemic in north-east coast of India. It is relatively common here, with nine specimens found.

### Discussion of Northern Bay of Bengal Nereididae

Pramanik et al. (2009) record 12 species of nereidid from Bangladesh: *Dendronereides heteropoda*, *Dendronereis aestuarina*, *Dendronereis arborifera*, *Lycastis indica*, *Namanereis quadraticeps*, *Nereis caudata*, *Nereis falcaria*, *Nereis lamellosa*, *Nereis mossambica*, *Nereis operta*, *Tylonereis bogoyawlenskyi* (sic) and *Tylonereis fauveli*. Alam et al. (1996) add *Perinereis nuntia* as well as *Lycastonereis indica* to this list.

The collections from Myanmar in the Natural History Museum, London, include: NHMUK ANEA 1933.3.18.43, NHMUK ANEA 1937.1.4.16-42 *Ceratonereis burmensis* Monro, 1937, Types; NHMUK ANEA 1931.6.22.70, NHMUK ANEA 2014.7 *Namalycastis abiuma* species group; NHMUK ANEA 1931.6.22.67-69 *Namalycastis multiseta* Glasby, 1999, Types; NHMUK ANEA 1931.6.22.71-73 *Neanthes meggitti* Monro, 1931, Types; NHMUK ANEA 1933.3.18.5-14, NHMUK ANEA 1935.1.31.6, NHMUK ANEA 1935.1.31.7-8 *Nereis falcaria*; NHMUK ANEA 1933.3.18.15-16 *Nereis* sp.; NHMUK ANEA 1933.3.18.1-4, NHMUK ANEA 1937.3.10.10-11 *Nereis zonata*; NHMUK ANEA 1937.3.10.12 *Perinereis cultrifera*?; NHMUK ANEA 1937.1.4.43-44 *Perinereis nuntia*; NHMUK ANEA 1932.11.25.5, NHMUK ANEA 1937.1.4.45-66 *Perinereis singaporiensis*; NHMUK ANEA 1932.11.25.2-3, NHMUK ANEA 1933.3.18.44-46, NHMUK ANEA 1935.1.31.16-18, NHMUK ANEA 1937.1.4.67-68 *Pseudonereis trimaculata*; NHMUK ANEA 1933.3.18.32-33 *Tylonereis bogoyawlenskyi*. Fauvel 1932 records *Leonnates jousseaumei* (from the Mergui Archipelago, shore collecting), *Lycastis meraukensis* (from Mergui), *Nereis onychophora* (from the Jack and Una Islands, Mergui Archipelago, shore collecting), *Perinereis cultrifera* var. *helleri* (from Mergui), *Perinereis singaporiensis* (from the Jack and Una Islands, shore collecting) and *Tylonereis fauveli* (from Mergui harbour, 7 fathoms).

Chandra and Chakraborty (2008), Das et al. (2009), Khan (2003), Misra (1999), Mitra and Misra (2010), Nesemann et al. (2007), Paul and Nandi (2003) and Sarkar et al. (2005) record 18 species (and one variety) of nereidid from coasts, estuaries, rivers and wetlands in West Bengal: *Ceratonereis burmensis*, *Dendronereides gangetica*, *Dendronereides heteropoda*, *Dendronereis aestuarina*, *Dendronereis dayi*, *Ganganereis sootai*, *Lycastonereis indica*, *Namalycastis fauveli*, *Namalycastis indica*, *Namalycastis meraukensis*, *Neanthes chilkaensis*, *Neanthes chingrighattensis*, *Neanthes glandicincta*, *Neanthes meggitti*, *Perinereis cavifrons*, *Perinereis cultrifera*, *Perinereis nigropunctata*, *Perinereis nuntia*, *Perinereis nuntia* var. *typica*.

Misra (1999), Mitra et al. (2010), Rao (1995) and Soota and Rao (1977) record 17 species of nereidid from coasts, estuaries, the Baitarani River and Chilka Lake in Odisha: *Ceratonereis burmensis*, *Dendronereides gangetica*, *Dendronereides heteropoda*, *Dendronereis aestuarina*, *Dendronereis dayi*, *Leonnates persica*, *Lycastonereis indica*, *Namalycastis fauveli*, *Namalycastis indica*, *Neanthes chingrighattensis*, *Nereis (Neanthes) chilkaensis*, *Nereis (Neanthes) glandicincta*, *Neanthes glandicincta*, *Nereis (Neanthes) reducta*, *Nereis (Neanthes) willeyi*, *Nereis (Nereis) persica*, *Perinereis cultrifera* and *Perinereis nigropunctata*.

Many of these species have had their names changed for taxonomic reasons, or are otherwise worthy of comment.

The genus *Ceratonereis* has been revised by Hartmann-Schröder (1985), who places the species *Ceratonereis burmensis* in the subgenus *Composetia*. *Composetia* has now been raised to generic level, but more work is needed on this grouping (Bakken and Wilson 2005).

*Leonnates jousseamei* has been synonymised with *Leonnates indicus* by Qiu and Qian (2000), who also correct *Leonnates persica* to *Leonnates persicus*.

Glasby (1999) states that *Namanereis quadraticeps* is restricted to the Subantarctic and temperate shores of the Southern hemisphere, and Glasby et al. (2009) refer it to the *Namanereis quadraticeps* (Blanchard in Gay, 1849) species group.

Glasby (1999) accepts *Lycastis indica* as a member of the genus *Namalycastis*, and also places *Namalycastis meraukensis* in the *Namalycastis abiuma* (Grube, 1872) species group.

*Nereis falcaria* was reduced to a subspecies of *Nereis jacksoni* by Hartmann-Schröder (1974) but the two species were separated again by Wu et al. (1981, see Wu et al. 1985). Wilson (1984) accepts *Nereis caudata* as a member of the genus *Neanthes*. Most members of the genera *Neanthes* and *Nereis* need to have their type specimens compared with the descriptions in Bakken and Wilson (2005) before their generic placement can be confirmed.

*Perinereis nuntia* has been studied by Glasby and Hsieh (2006), Wilson and Glasby (1993) and Yousefi et al. (2011), and as Alam et al. (1996) did not give a full description of their specimens, it would be better to refer them to the *Perinereis nuntia* (Savigny, 1818) species group. The specimen 1937.1.4.43-44 from Maungmagaun, Myanmar, has been studied and identified as *Perinereis nuntia* as defined by Glasby and Hsieh (2006). *Perinereis helleri* was kept separate from *Perinereis cultrifera* by Hutchings et al. (1991), but was synonymised with it by Khlebovich (1996). Problems with species of *Perinereis* were also discussed by Muir and Bamber (2008).

The very similar species *Pseudonereis trimaculata* and *Pseudonereis variegata* have been kept separate by Bakken (2007) and Villalobos-Guerrero and Tovar-Hernández (2013). Most characters seem to overlap completely, but in *Pseudonereis trimaculata* the dorsal cirrus, rather being sub-terminal, is attached to the notopodium terminally from about chaetiger 40, and the ventral ligule of the neuropodium is 0.5-0.8 times as long as the acicular ligule in anterior chaetigers. In *Pseudonereis variegata* only the last few dorsal cirri are attached terminally, and the ventral ligule of the neuropodium is as long as the acicular ligule in anterior chaetigers (it is as long as the acicular ligule in posterior chaetigers in both species). It is not surprising, therefore, that Monro labelled sample 1932.11.25.2-3 in the NHM as *Pseudonereis trimaculata* = *variegata*. The four samples from Myanmar have now been re-examined, and while some are definitely *Pseudonereis trimaculata*, others have the longer ventral ligule of the neuropodium in anterior chaetigers of *Pseudonereis variegata* while also having the dorsal cirrus attached to the notopodium terminally in the last quarter of the body. We are treating all the Myanmar specimens as *Pseudonereis trimaculata*, but mentioning both species in the key.

The relevant species mentioned above can be keyed out as follows, but any identifications must be checked against reliable descriptions as many other species are known from the Bay of Bengal and other Indo–Pacific areas.

**Table:** 

1	Three pairs of tentacular cirri. Paragnaths absent from pharynx	*Lycastonereis indica* Rao, 1981
–	Four pairs of tentacular cirri. Paragnaths present or absent	2
2	Paragnaths absent from pharynx	3
–	Paragnaths present on pharynx	16
3	Branchiae present dorsally on some notopodia	4
–	Branchiae absent from notopodia	8
4	Cluster of branchial filaments below dorsal cirrus on about segments 8 to 40	5
–	Dorsal cirrus develops into bipinnate branchia on some middle segments	6
5	Branchiae as row of simple filaments, later developing into two whorls of filaments, on segments 10–38	*Dendronereides gangetica* Misra, 1999
–	Cluster of branched branchiae below dorsal cirrus on segments 8 to 40	*Dendronereides heteropoda* Southern, 1921
6	Chaetae include homogomph and heterogomph spinigers. Papillae present on both rings of pharynx	*Dendronereis dayi* Misra, 1999
–	Chaetae all homogomph spinigers. Papillae absent or only on oral ring of pharynx	7
7	Pharyngeal papillae absent. All branchiae bipinnate. Anterior neuropodia with 5–6 lobes	*Dendronereis arborifera* Peters, 1854
–	Papillae present on oral ring of pharynx. First three pairs of branchiae pectinate, the rest bipinnate. Anterior neuropodia with 10–12 lobes and an inferior ligule (number decreasing posteriorly)	*Dendronereis aestuarina* Southern, 1921
8	Parapodia clearly biramous. All chaetae spinigers	10
–	Parapodia clearly biramous. Spinigers and homogomph falcigers present	9
–	Parapodia with no deep separation between notopodium and neuropodium. Spinigers and heterogomph falcigers present	12
9	Falcigers present on all neuropodia	*Leonnates indicus* Kinberg, 1866
–	Falcigers not present on anterior neuropodia	*Leonnates persicus* Wesenberg-Lund, 1949
10	Neurochaetae include homo-, sesqui- and heterogomph spinigers	*Ganganereis sootai* Misra, 1999
–	All chaetae homogomph spinigers	11
11	Neuropodia trilobed anteriorly, bilobed posteriorly	*Tylonereis bogoyawlenskyi* Fauvel, 1911
–	All neuropodia bilobed	*Tylonereis fauveli* Southern, 1921
12	Body widest mid-anteriorly (chaetigers 9–20). Sub-neuroacicular chaetae heterogomph spinigers and falcigers	15
–	Body with uniform width anteriorly, tapering in far posterior region	13
13	Sub-neuroacicular chaetae heterogomph falcigers and heterogomph spinigers	14
–	Sub-neuroacicular chaetae heterogomph falcigers	*Namanereis quadraticeps* (Blanchard in Gay, 1849) species group
14	Prostomium 1.3–2.3× wider than long. Usually less than 10 sesquigomph spinigers in neuropodial supra-acicular fascicle in midbody	*Namalycastis abiuma* (Grube, 1872) species group
–	Prostomium 2.4× wider than long or even wider. 10-30 sesquigomph spinigers in neuropodial supra-acicular fascicle in midbody	*Namalycastis multiseta* Glasby, 1999
15	Antennae minute, not reaching tip of palpophore. Heterogomph falcigers with boss extremely prolonged. Jaw with 2-3 subterminal teeth + 2-4 ensheathed proximally	*Namalycastis fauveli* Rao, 1981
–	Antennae more or less reaching tip of palpophore. Heterogomph falcigers with boss not prolonged. Jaw with 2-5 subterminal teeth + 3-5 ensheathed proximally	*Namalycastis indica* (Southern, 1921)
16	Groups V, VI, VII and VIII with no paragnaths	*Composetia burmensis* (Monro, 1937)
–	Groups V, VI, VII and VIII with paragnaths	17
17	Group VI = at least one transverse paragnath	18
–	All paragnaths in the shape of cones or small dots	22
18	Group VI = one transverse bar	19
–	Group VI = two transverse bars	*Perinereis singaporiensis* Grube, 1878
–	Groups V and VI have a continuous row of transverse bars	*Perinereis nuntia* (Savigny, 1818) species group
19	Group V absent	*Perinereis cavifrons* Ehlers, 1920
–	Group V = 1-3 paragnaths	20
20	Group I = 1-3 paragnaths	21
–	Group I = 6-12 paragnaths in an irregular group	*Perinereis nigropunctata* (Horst, 1889)
21	Groups II-IV arranged in clusters	*Perinereis cultrifera* (Grube, 1840)
–	Groups II-IV arranged in regular comb-like rows	33
22	One simple falciger present in posterior notopodia	*Nereis onychophora* Horst, 1918
–	Compound notopodial falcigers present posteriorly	23
–	Notopodial falcigers absent posteriorly	26
23	Groups VII and VIII as a single row except in juveniles where it may be double	*Nereis falcaria* (Willey, 1905)
–	Groups VII and VIII as an irregular band two to four deep	24
24	Apices of notopodial falcigers with 1-3 large teeth	*Nereis persica* Fauvel, 1911
–	Apices of notopodial falcigers smooth or lightly serrate	25
25	Group I = 0, Group III = 5 in single transverse row, Groups VII and VIII = a broad band with an anterior row of large paragnaths and two to three posterior rows of smaller ones	*Nereis jacksoni* Kinberg, 1866
–	Group I = 1-3, Group III = 12-22 in a transverse group, Groups VII and VIII = 2 rows, the posterior with smaller, more numerous paragnaths	*Nereis zonata* Malmgren, 1867
–	Group I = 1-3, Group III = about three rows totalling 20-30, Groups VII and VIII = three or four irregular rows	*Nereis lamellosa* Ehlers, 1868
26	Falcigerous chaetae entirely absent	*Neanthes chingrighattensis* Fauvel, 1932
–	Some falcigers present in neuropodia	27
27	Paragnaths of basal ring forming a continuous band which is broad ventrally	28
–	Paragnaths of V and VI separate, Groups VII and VIII forming a band 2-4 deep	*Neanthes willeyi* (Day, 1934)
–	Paragnaths of V absent, Groups VII and VIII forming a band of one to three rows	30
28	Group I = 1 paragnath	*Neanthes operta* Stimpson, 1856
–	Group I = 4-12 paragnaths, which may be small	29
29	Groups V, VI, VII and VIII form a complete broad band of several rows of paragnaths	*Neanthes caudata* (Chiaje, 1841)
–	Group V = 4-6 rather large paragnaths, Group VI = 5-6 paragnaths in a round cluster, Groups VII and VIII form 3-4 irregular rows of very large and small cones	*Neanthes meggitti* Monro, 1931
30.	Group VI = 1 paragnath	31
–	Group VI = several paragnaths (may be minute)	32
31	Group VI = 1 paragnath; Group IV = 6-12 large paragnaths	*Neanthes glandicincta* (Southern, 1921)
–	Group VI = 1 large, oval, paragnath; Group IV = a wedge of about 20 paragnaths	*Neanthes mossambica* Day, 1957
32	Group I = 6-10 paragnaths, Group II = 18-20 paragnaths, Group III = a transverse band of 3-4 rows of paragnaths	*Neanthes chilkaensis* (Southern, 1921)
–	Group I = 1 paragnath, Group II = 6 paragnaths, Group III = 11 paragnaths	*Neanthes reducta* (Southern, 1921)
33	Group V = 1-3 paragnaths. Neuropodia of anterior chaetigers with ventral ligule shorter than the acicular ligule. Dorsal cirrus attached terminally in the last quarter of the body	*Pseudonereis trimaculata* Horst, 1924
–	Group V = 1 paragnath. Neuropodia of anterior chaetigers with ventral ligule as long as the acicular ligule. Dorsal cirrus terminal in the last few chaetigers only	*Pseudonereis variegata* (Grube, 1857)

### Lumbrineridae

The Lumbrineridae used to be regarded as part of the family Eunicidae (e.g. Fauvel (1953), Day (1967)), but is now regarded as a separate family alongside the Eunicidae and various others in the Order Eunicida (see George and Hartmann-Schröder (1985), Carrera-Parra (2006a)).

The pharynx is usually not everted in preserved material, but the maxillae may be seen by making a lateral cut (not a mid-ventral cut as used for nereidids) backwards from the side of the mouth, cutting through the side of the pharynx as well as the body wall for several segments, and folding the resulting flap to the side to reveal the complete jaw apparatus. If it is necessary to dissect the pharynx in this way, the anterior chaetae should be studied first.

### *Gesaneris malayensis* (Rullier, 1969)

**Figure 5.**
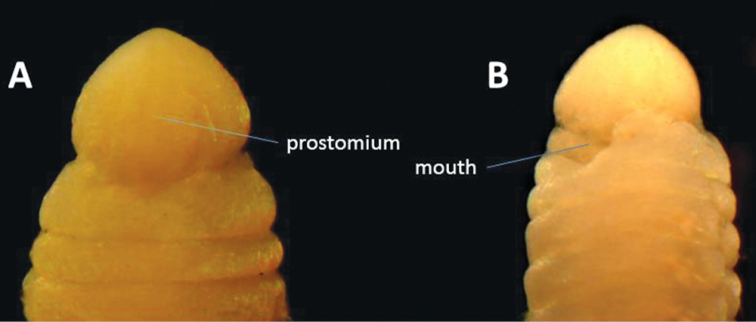
Anterior end of *Gesaneris malayensis*. **A** Dorsal view **B** Ventral view.

Six specimens of this species (Fig. 5) were found. The species, originally published as *Lumbriconereis malayensis*, was redescribed by Carrera-Parra (2006a) and transferred to a new genus. It is a new record for Bangladesh according to Pramanik et al. (2009). It is very similar to the description of *Eranno papillifera* (Fauvel, 1918) by Oug (2002). This latter species was also first published as a *Lumbriconereis* species. The most important difference is that in *Gesaneris* the maxillary apparatus has four pairs of maxillae, whereas in *Eranno* there are five pairs of maxillae.

### Discussion of Northern Bay of Bengal Lumbrineridae

Previous records of lumbrinerids in Bangladesh are *Lumbrinereis heteropoda heteropoda* (Marenzeller, 1879) and *Lumbrinereis tetraura* (Schmarda, 1861), recorded by Alam et al. (1996) and Pramanik et al. (2009).

The collections of the Natural History Museum, London, contain a previously unpublished specimen (NHMUK ANEA 1937.3.10.15) collected by Prof. F.J. Meggitt at Maungmagaun, Myanmar, which can be identified using the key in Fauvel (1953) as *Lumbriconereis sphaerocephala*. This species has not been re-studied by recent taxonomists but it is similar to *Lumbrineris inflata* Moore, 1911 (see Carrera-Parra 2006b).

Das et al. (2009) record *Lumbrineris heteropoda*, *Lumbrineris polydesma* and *Lumbrineris notocirrata* from West Bengal.

In Odisha, the species *Lumbrineris heteropoda*, *Lumbrineris notocirrata* and *Lumbrineris polydesma* have been recorded from coasts and estuaries, while *Lumbrineris polydesma* and *Lumbrineris simplex* were found in Chilka Lake (Misra 1999, Mitra et al. 2010, Rao 1995).

*Lumbrineris heteropoda* has been transferred to the genus *Kuwaita* by Carrera-Parra and Orensanz (2002) and to the genus *Scoletoma* by Budaeva (2005). The species appears to have a wide geographic range (Sea of Okhotsk to Red Sea) and may well be sub-divided after further study, but for the moment we shall accept the name *Kuwaita heteropoda*, as Carrera-Parra and Orensanz (2002) studied a specimen from Japan, the type locality.

*Lumbrineris tetraura* is accepted as a member of the genus *Lumbrineris* by e.g. George and Hartmann-Schröder (1985) who say it is a cosmopolitan species, but Diaz-Castaneda and Rodriguez-Villanueva(1998), place it in the genus *Scoletoma*, the members of which only have simple hooded hooks, whereas *Lumbrineris* species possess both simple and composite hooded hooks. *Lumbrineris polydesma* may also be better placed in the genus *Scoletoma*, but it is not formally transferred here because the type specimens have not been studied.

*Lumbrineris simplex*, having no hooked chaetae and no antennae, is better placed in the genus *Arabellonereis*.

*Lumbrineris notocirrata* has been transferred to the genus *Ninoe* by Fauchald (1970), because of the presence of small branchiae.

The species *Lumbriconereis pseudobifilaris* Fauvel, 1932, has been recorded from 250 fathoms (about 450 m) depth off Akyab, Myanmar (Fauvel 1932), and from a sewage-fed fish culture pond near Calcutta, West Bengal, India (Mitra and Roy 2010). This species has no hooked chaetae but it does have two very long maxillary carriers. It is not included in the key because these characters make it a member of the family Oenonidae.

*Lumbrinereis* and *Lumbriconereis* are incorrect spellings of the generic name *Lumbrineris*. The northern Bay of Bengal ecoregion species of Lumbrineridae can be keyed out as follows, but any identifications must be checked against reliable descriptions as many other species are known from the Bay of Bengal and other Indo–Pacific areas.

**Table:** 

1	Three antennae present	*Kuwaita heteropoda* (Marenzeller, 1879)
–	Antennae absent	2
2	Hooded hook chaetae absent	*Arabellonereis simplex* (Southern, 1921)
–	Hooded hook chaetae presen	3
3	Simple hooded hooks only	4
–	Compound hooded hooks present anteriorly, as well as simple hooded hooks along the body	*Lumbrineris sphaerocephala* (Schmarda, 1861)
4	Lateral mouth pads present. Branchiae present in posterior part of body, as a small dorsal knob or transparent vesicle, a little above the base of the parapodium	*Ninoe notocirrata* (Fauvel, 1932)
–	Lateral mouth pads absent. Branchiae absent	5
5	Hooks start about chaetiger 29 or 30	*Lumbrineris polydesma* Southern, 1921
–	Hooks start about chaetiger 1-4	6
6	Maxillary apparatus with five pairs of maxillae. Maxilla IV completely pigmented. Hooks with entire main tooth and crest of smaller teeth above it	*Scoletoma tetraura* (Schmarda, 1861)
–	Maxillary apparatus with four pairs of maxillae. Maxilla IV with whitish central area. Hooks with furcate main tooth and crest of smaller teeth above it	*Gesaneris malayensis* (Rullier, 1969)

## Glyceridae

A posterior fragment of a glycerid was found in this collection, but it is unidentifiable to species. It is, however, a new record for Bangladesh because the fragment appears to bear branchiae on the parapodia, whereas *Glycera lancadivae* Schmarda, 1861, the only species in Pramanik et al. (2009), does not. Böggemann (2002) says that *Glycera lancadivae* is a *nomen dubium*, but similar to *Glycera brevicirris* (known from Sri Lanka and the Andaman Sea) and *Glycera tesselata* (nearest known localities Xisha Islands and Madagascar).

The collections of the Natural History Museum, London, contain a specimen of *Glycera cinnamomea* (NHMUK ANEA 1938.5.7.45) which was collected from Investigator station 549, at a depth of 24 fms (43.89 m), off Mergui Harbour (= Myeik, near the mouth of the Tanintharyi river, Myanmar), identified by Böggemann (2002). Fauvel (1932) records *Glycera cirrata* (from off Tenasserim, Burma, 50 fathoms (91.44 m)). Böggemann (2002) says this species is mixture of *Glycera brevicirris* (known from Sri Lanka and the Andaman Sea) and *Glycera americana* (nearest known localities on the coasts of South America).

Das et al. 2009 record *Glycera convoluta* and *Glycera rouxii* from West Bengal.

*Glycera convoluta*, *Glycera lancadivae*, *Glycera longipinnis*, *Glycera rouxii*, and *Glycera tesselata* have been reported by Misra (1999) and Mitra et al. (2010) from the coasts and estuaries of Odisha. According to the major revision by Böggemann (2002), *Glycera convoluta* is probably a junior synonym of *Glycera tridactyla*, *Glycera lancadivae* is a nomen dubium, *Glycera longipinnis* is a junior synonym of *Glycera sphyrabrancha*, *Glycera rouxii* is probably a junior synonym of *Glycera unicornis*, and *Glycera tesselata* is a good species. *Glycinde oligodon* Southern, 1921, has also been reported from the Chilka Lake, Odisha, as a glycerid by Rao (1995), but this species belongs to the family Goniadidae.

Böggemann (2002) accepts 14 species of glycerid from the Bay of Bengal area. The following key to these 14 species plus *Glycera tesselata* and *Glycera unicornis* (not previously recorded from the Bay of Bengal) is derived from Böggemann (2002), which contains full descriptions of these and many other species from the Indo–Pacific region.

**Table:** 

1	All parapodia uniramous, notopodia absent. Ailerons rod-like	*Hemipodia simplex* (Grube, 1857)
–	Parapodia biramous after (usually) first two, notopodia with simple capillary chaetae. Ailerons with a more or less triangular or deeply incised base	2
2	Proboscideal papillae without terminal fingernail structure	3
–	Proboscideal papillae with terminal fingernail structure	7
3	One postchaetal lobe in all parapodia	4
–	Two postchaetal lobes at least on parapodia of mid-body	6
4	In mid-body, notopodial prechaetal lobes shorter than neuropodial lobes. Branchiae absent	*Glycera lapidum* Quatrefages, 1866
–	In mid-body, prechaetal lobes of about same length or notopodial lobes longer. Branchiae present or absent	5
5	Digitiform proboscideal papillae without ridges. Ailerons with deeply incised bases. Simple digitiform branchiae situated termino-dorsally on parapodia	*Glycera sphyrabrancha* Schmarda, 1861
–	Conical proboscideal papillae with about 5–20 transverse ridges. Ailerons with slightly arched bases. Branchiae absent	*Glycera oxycephala* Ehlers, 1887
6	Ailerons with deeply incised bases. Postchaetal lobes short, rounded. Branchiae absent	7
–	Ailerons with interramal plate and triangular bases. Parapodia of mid-body with slender triangular notopodial and distinctly shorter rounded neuropodial postchaetal lobes. Retractile branchiae situated medially on anterior side of parapodia	8
7	Digitiform proboscideal papillae with straight, median, longitudinal ridge	*Glycera tesselata* Grube, 1863
–	Digitiform proboscideal papillae with about 6-20 transverse ridges	*Glycera brevicirris* Grube, 1870
8	Parapodia with slender triangular notopodial and distinctly shorter, rounded, neuropodial postchaetal lobes; simple digitiform retractile branchiae	*Glycera nicobarica* Grube, 1868
–	Parapodia with two slender triangular postchaetal lobes of about same length or notopodial lobes only slightly longer than neuropodial lobes; digitiform retractile branchiae with 1-2 rami	*Glycera unicornis* Savigny, 1818
9	Parapodia of mid-body with two slender triangular postchaetal lobes of about same length	10
–	Parapodia of mid-body with slender triangular notopodial and shorter, more or less rounded, neuropodial postchaetal lobes	11
10	Parapodia without branchiae	*Glycera onomichiensis* Izuka, 1912
–	1–5 digitiform branchial rami situated dorsally on parapodial bases	*Glycera cinnamomea* Grube, 1874
11	In mid-body and posterior parapodia neuropodial postchaetal lobes more or less rounded. Simple digitiform branchiae situated termino-dorsally on parapodia	12
–	In posterior parapodia neuropodial postchaetal lobes as long as notopodial lobes and equally slender triangular. Simple digitiform branchiae situated medio-dorsally on parapodia	*Glycera posterobranchia* Hoagland, 1920
12	All biramous parapodia with two postchaetal lobes. Proboscideal papillae with long, medium or short stalk	13
–	In anterior parapodia only one, medially inserted slender triangular postchaetal lobe. Proboscideal papillae with short stalk	*Glycera macrobranchia* Moore, 1911
13	Proboscideal papillae with long stalk	14
–	Proboscideal papillae with medium-length or short stalk	15
14	Stalk without ridges. Ailerons with pointed triangular bases	*Glycera alba* (Müller, 1776)
–	Stalk with numerous ridges. Ailerons with triangular bases	*Glycera natalensis* Day, 1957
15	Proboscideal papillae with short stalk. Prostomium consisting of about 11–15 rings. Ailerons with triangular bases	*Glycera tridactyla* Schmarda, 1861
–	Proboscideal papillae with medium-length stalk. Prostomium consisting of about 19–28 rings. Ailerons with pointed triangular bases	*Glycera africana* Arwidsson, 1899

### Nephtyidae
*Micronephthys oligobranchia* (Southern, 1921)

**Figure 6.**
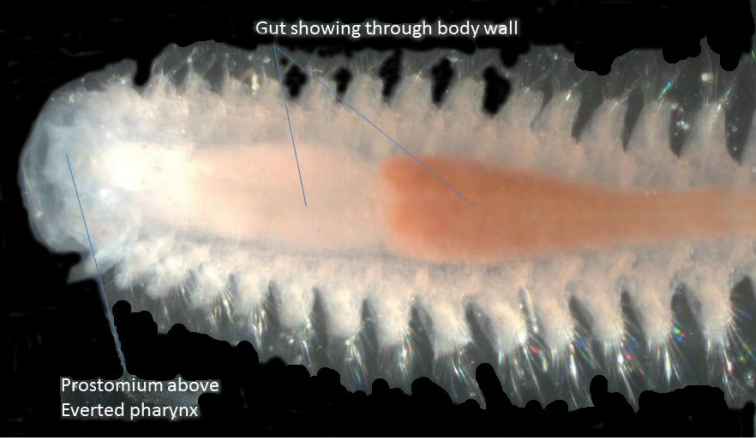
Dorsal view of semi-transparent specimen of *Micronephtys oligobranchia* with partially everted pharynx.

The single 6.5 mm long worm found (Fig. 6) is probably this species, which has been well described by Imajima (1987) under the name *Nephtys oligobranchia*. The species has been transferred to the genus *Micronephthys* by Dnestrovskaya and Jirkov (2010), although they say the family needs to be fully reviewed. Glasby et al. (2009) list *Nephtys oligobranchia* as a freshwater- and saltwater-tolerant species, found in the Palaearctic and Oriental regions inhabiting lake/river freshwater, estuary and coastal lagoons (fresh-brackish) including supra-littoral areas and inland lakes. Shen and Qi (1982) say it is “favored in normal or rich trophic waters”, as opposed to over-trophic or polluted waters.

### Discussion of Northern Bay of Bengal Nephtyidae

*Nephtys oligobranchia* is the only nephtyid recorded by Pramanik et al. (2009) from Bangladesh. It is also recorded by Fauvel (1932) from Mergui, Myanmar.

*Nephtys oligobranchia* and *Nephtys polybranchia* Southern, 1921, have both been recorded from West Bengal (Misra 1999) and the Ganges river system (Nesemann et al. 2007). Das et al. (2009) record *Nephtys dibranchis* and *Nephtys oligobranchia*. *Nephtys dibranchis* was placed in the genus *Aglaophamus* by Hartman (1950).

*Nephtys oligobranchia* is recorded from the Baitarani River, Odisha, by Misra (1999). *Nephtys oligobranchia* and *Nephtys polybranchia* are both recorded from the Chilka Lake (Misra 1999, Mitra et al. 2010).

The northern Bay of Bengal ecoregion species of Nephtyidae can be keyed out as follows, but any identifications must be checked against reliable descriptions as many other species are known from the Bay of Bengal and other Indo–Pacific areas.

**Table:** 

1	Interramal cirri (branchiae) large and curving in towards the body, especially anteriorly	*Aglaophamus dibranchis* (Grube, 1878)
–	Interramal cirri poorly developed	2
2	Interramal cirri absent from posterior half of body	*Micronephtys oligobranchia* (Southern, 1921)
–	Interramal cirri continuing more or less to end of body	*Nephtys polybranchia* Southern, 1921

### Capitellidae

Specimens of Capitellidae, having no head appendages and usually no obvious parapodial lobes, can easily be mistaken for oligochaetes (e.g. Stephenson 1908, 1910). Without studying the reproductive system in detail, the most useful distinguishing character is the presence of hooked chaetae with a terminal hood covering the hook, which are found on the abdomen of capitellids (absent from oligochaetes).

### *Heteromastus filiformis* sensu Day

**Figure 7.**
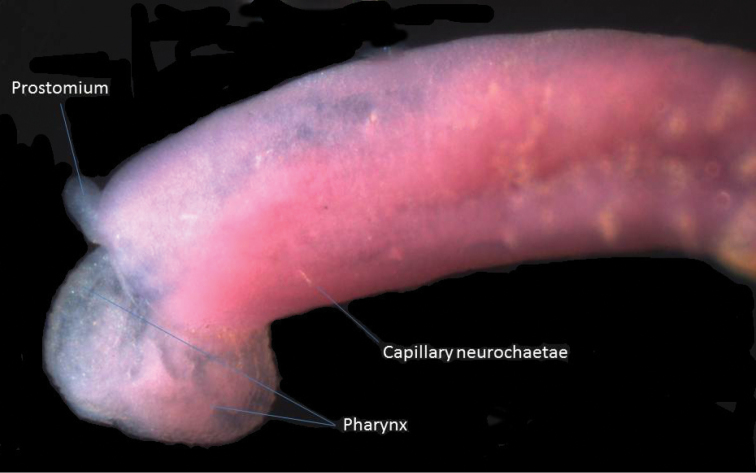
Lateral view of anterior end of *Heteromastus filiformis* with everted pharynx.

Green (2002) gives a key to members of the genus *Heteromastus* found in the Indian Ocean. Unfortunately, there are variations in the descriptions of *Heteromastus filiformis* (Claparède, 1864) specimens described by different people. The three specimens found in this collection (Fig. 7), having expanded neuropodial lobes in the posterior abdomen and abdominal hooded hook chaetae with three teeth above the main fang, are more like those described by Day (1967) than those described by Hutchings and Rainer (1981), which had expanded notopodial lobes in the posterior abdomen and abdominal hooded hook chaetae with 11–13 teeth above the main fang. The type locality of *Heteromastus filiformis* is in the Mediterranean (see Green 2002), so Day’s specimens from South Africa could be a different species to Claparède’s. Glasby et al. (2009) list *Heteromastus filiformis* as freshwater tolerant, but say that multiple species may have been reported under this name.

This is a new record according to Pramanik et al. (2009).

### Discussion of Northern Bay of Bengal Capitellidae

Pramanik et al. (2009) give *Dasybranchus caducus* as the only capitellid species they know from Bangladesh.

The Natural History Museum, London, has a specimen of *Notomastus* near *latericeus* Sars, 1851 sensu Green 2002 (NHMUK ANEA 1933.3.18.71) and the type specimens of *Parheteromastus tenuis* Monro, 1937, from Maungmagaun, Myanmar (NHMUK ANEA 1937.1.4.151-163).

*Matla bengalensis* Stephenson, 1908, was described as a new genus and species of oligochaete from West Bengal, but is now (Stephenson 1910) recognised as a capitellid polychaete similar to *Capitella capitata*. Alam et al. (2010), Chandra and Chakraborty (2008), Das et al. (2009), Misra (1999), Mitra and Misra (2010) and Sarkar et al. (2005) report *Barantolla sculpta* Southern, 1921, *Capitella capitata* (Fabricius, 1780), *Mastobranchus indicus* Southern, 1921, and *Parheteromastus tenuis* Monro, 1937, from coasts, mangroves, lakes and rivers of West Bengal.

In Odisha, five species of capitellid have been reported (Rao 1995), all of them from the Chilka Lake – *Barantolla sculpta*, *Capitella capitata*, *Heteromastus similis*, *Notomastus latericeus* and *Pulliella armata*. Magalhães and Bailey-Brock (2012) consider *Pulliella* to be a junior synonym of *Scyphoproctus*.

There follows a key to the reported species of capitellid from the Northern Bay of Bengal ecoregion, but any identifications must be checked against reliable descriptions as many other species are known from the Bay of Bengal and other Indo–Pacific areas. In particular, Green (2002) suggested earlier records of *Heteromastus similis* may be suspect. Also, as noted above, the type locality of *Capitella capitata* is West Greenland (Blake 2009). In both these cases further taxonomic work is needed, including the careful study of type specimens and probably comparisons of DNA, to see whether the Bay of Bengal specimens have been misidentified and actually represent new species (see, for example, Blake (2009), Blake et al. (2009), Wu et al. (1991)).

Most keys to capitellid genera start with the number of segments in the thorax. There may be a sudden change in size or shape of the segments at the start of the abdomen, but in many cases it is easier to make a temporary whole mount of the specimen and count the segments with capillary chaetae.

**Table:** 

1	Posterior abdominal segments with stout acicular chaetae; pygidium with two stout, conical, diverging anal cirri	*Scyphoproctus armatus* (Fauvel, 1929)
–	Posterior abdominal segments with hooded hook chaetae; anal cirri absent	2
2	4-7 anterior segments with capillary chaetae	3
–	11-13 anterior segments with capillary chaetae	6
3	Thorax with 9 segments, all with chaetae	*Capitella capitata* (Fabricius, 1780)
–	Thorax with 12 segments, the first achaetous	4
4	4 anterior segments with capillary chaetae	*Parheteromastus tenuis* Monro, 1937
–	5 anterior segments with capillary chaetae	5
–	6 anterior segments with capillary chaetae	*Barantolla sculpta* Southern, 1921
5	Abdominal hooks with longer anterior shaft than posterior shaft (node posterior to middle of shaft), two rows of teeth above main fang; posterior abdomen with expanded notopodial lobes	*Heteromastus similis* Southern, 1921
–	Abdominal hooks with longer posterior shaft than anterior shaft (node anterior to middle of shaft), three teeth above main fang; posterior abdomen with expanded neuropodial lobes	*Heteromastus filiformis* (Claparède, 1864) sensu Day, 1967
6	11 anterior segments with capillary chaetae	7
–	13 anterior segments with capillary chaetae	*Dasybranchus caducus* (Grube, 1846)
7	Abdomen with anterior two or more segments with mixed fascicles of capillary chaetae and hooded hooks	*Mastobranchus indicus* Southern, 1921
–	No segments with mixed chaetal fascicles	*Notomastus* near *latericeus* Sars, 1851

## General discussion

Of the seven taxa identified above, five are new records for Bangladesh. This shows that the polychaete fauna of Bangladesh is still not well known.

Some earlier records of polychaetes will have to be re-studied before the fauna list of the Bangladesh area is complete, as names will change for taxonomic or nomenclatural reasons. An example of this is the genus *Talehsapia*, reported from the Hooghly estuary and South 24-Parganas, West Bengal by Misra (1999) and Mitra and Misra (2010). The genus was placed in the new family Talehsapiidae Misra, 1999, but it is now known to fit into the pilargid subfamily Synelminae (see Salazar-Vallejo et al. 2001). More recently, *Talehsapia* has been synonymised with the genus *Hermundura* (see Glasby and Hocknull 2010).

It is notable that some species reported from Bangladesh have very wide reported distributions – the same species being reported from both Bangladesh and northern Europe may be the result of misidentification or an unrecognised cryptic species. It is also notable that Ghosh (2014) reports that two polychaete species first found in this ecoregion – *Asychis gangeticus* Fauvel, 1932 (family Maldanidae; type locality the Gangetic delta) and *Pseudopolydora kempi* (Southern, 1921) (family Spionidae; type locality a canal at Chingrighatta near Kolkata) – were not found by the Zoological Survey of India between 1984 and 1989, possibly due to the river flow being reduced by dams.

While the surface salinity in the open part of the Bay of Bengal oscillates from 32–34.5‰, in the coastal region it varies from 10–25‰ and at the river mouths the surface salinity decreases to 5‰ or even less (Banglapedia 2010). It is no surprise, therefore, that some of the species in this paper are listed by Glasby et al. (2009) as being freshwater tolerant.

Further work on the occurrance and abundance of the macrobenthic fauna and ecology of the Sitakunda coast, Chittagong, will be published in the future (Hossain and M. Belal in prep.).
